# Assessing CD36 and CD47 expression levels in solid tumor indications to stratify patients for VT1021 treatment

**DOI:** 10.1038/s41698-024-00774-9

**Published:** 2024-12-03

**Authors:** Suming Wang, Victor Zota, Melanie Y. Vincent, Donna Clossey, Jian Jenny Chen, Michael Cieslewicz, Randolph S. Watnick, James Mahoney, Jing Watnick

**Affiliations:** 1 Vigeo Therapeutics, Cambridge, MA USA; 2 Boston Children’s Hospital, Boston, MA USA

**Keywords:** Prognostic markers, Tumour biomarkers, Targeted therapies

## Abstract

Despite the development of cancer biomarkers and targeted therapies, most cancer patients do not have a specific biomarker directly associated with effective treatment options. We have developed VT1021 that induces the expression of thrombospondin-1 (TSP-1) in myeloid-derived suppressor cells (MDSCs) recruited to the tumor microenvironment (TME). Our studies identified CD36 and CD47 as dual biomarkers that can be used as patient stratifying tools and prognostic biomarkers for VT1021 treatment.

Through decades of development of cancer treatments, current treatment modalities can be divided into a limited number of categories, including surgery, radiation therapy, chemotherapy, hormone therapy, immunotherapy, stem cell transplant, and targeted therapy, which are administered as monotherapies or in combination^1^. To determine efficacious treatments for patients, cancer biomarkers are now being more broadly used. Despite some success in treating patients with currently available drugs that target specific biomarkers, most cancer patients do not have a specific biomarker that has been associated with effective treatment options. One therapeutic strategy that could significantly benefit from improved biomarker identification is immunotherapy, and, in general, therapies that target the tumor microenvironment (TME).

We have developed VT1021, a cyclic peptide derived from Prosaposin, that has shown single-agent efficacy in the clinic by inducing the expression of thrombospondin-1 (TSP-1) in the TME. The induction of TSP-1 in the TME has a multimodal therapeutic activity. Specifically, TSP-1 possesses anti-angiogenic, anti-tumorigenic, anti-inflammatory, and immunomodulatory activity. TSP-1 inhibits angiogenesis by inducing apoptosis in endothelial cells via downstream signaling from one of its cell surface receptors, CD36^2–5^. TSP-1 exerts its anti-tumorigenic activity by inducing the same apoptotic signaling cascade downstream from CD36 in tumor cells^3,6,7^. The anti-inflammatory activity of TSP-1 reprograms macrophages from M2 to M1 polarization via CD36^8–10^. TSP-1 also binds to the cell surface receptor CD47, also known as Integrin Associated Protein (IAP)^11^. By binding to CD47 on tumor cells, TSP-1 blocks the “do not eat me” signal induced by CD47 binding to SIRPα on macrophages^12^. The combined activity of reprogramming macrophages from M2 to M1 polarization and blocking CD47-SIRPa signaling stimulates macrophage phagocytosis and killing of tumor cells. Finally, TSP-1 converts immunosuppressive, or “cold”, TMEs to immune-active, or “hot”, TMEs by inducing cell death of regulatory T cells (Tregs) via CD36, and by preventing T cell inactivation by inhibiting VEGFR signaling via binding to CD47^13,14^.

Because VT1021 does not target a specific mutation in tumor cells but rather induces TSP-1 that inhibits tumor growth via binding to CD36 and CD47 on the surface of tumor cells, it is critical to identify patients that express sufficient levels of these receptors to maximize its anti-tumor activity.

CD36 is a fatty acid translocase^15,16^ that stimulates tumor cell proliferation by increasing fatty acid uptake and lipid metabolism^17^. The function of CD36 in cancers has been well studied in multiple reports^17–19^. CD36 has been shown to be expressed in multiple human cancer cell lines including those derived from pancreatic, ovarian, breast, and prostate cancer^7,20^. It has been reported that the CD36 gene is amplified and mutated in different cancers^21^. CD36 expression was also found to be positively correlated with disease progression, and worse prognosis in multiple cancers^18,19^, including brain cancer^22^. CD36 also contributes to cancer cell metastasis^23–25^, as CD36 expression is increased in metastatic tumors compared to primary tumors^7^. CD36 is also associated with cancer stem cells (CSCs), promoting CSCs proliferation, and maintaining stemness^22,26–29^. It has been reported that tumor cells upregulate CD36 to resist different treatments^18^. Last but not least, CD36 plays a key role in immune evasion, maintaining immunosuppressive regulatory T cells and pro-tumorigenic M2 macrophages^9,30,31^, suppressing tumor-infiltrating CD8^+^ T cells^32,33^.

CD47 is a membrane receptor glycoprotein ubiquitously expressed on both healthy and malignant cells^34^. On red blood cells, CD47 serves as a “do not eat me” signal marker to inhibit red blood cell phagocytosis^35^. CD47 was initially identified as a potential tumor marker for ovarian cancer^36^, and later studies found CD47 was overexpressed in various types of cancer^37–39^. CD47 was also considered as a tumor phagocytosis checkpoint marker and various cancers utilize it as an immune evasion tool^40,41^. It has been reported that CD47 signaling also participates in macrophage recruitment into tumors^42^ and polarizing the phenotype of macrophages in a human glioblastoma model^43^. Monoclonal antibodies against CD47 inhibit tumor growth and prolong survival in mouse models of GBM^44^. Moreover, CD47 is also associated with CSCs, stimulating CSCs to differentiate into mature cells^37^. High levels of CD47 expression are prognostic indicators of poor outcomes for cancer patients^38,39,45–48^.

To date, VT1021 has finished a phase I clinical trial to test its safety and preliminary clinical efficacy. In the recurrent Glioblastoma (rGBM) cohort of the expansion portion, 10 out of 22 evaluable patients (45%) showed clinical benefits after VT1021 treatment (as measured by complete or partial response and prolonged stable disease)^49,50^. Consistent with its established biological activity, TSP-1 expression was significantly increased in patient biopsies after VT1021 treatment. We found that the patient response rate was strongly correlated with the level of CD36 and CD47 expression on the surface of tumor cells. To investigate if CD36 and CD47 can be more widely used as biomarkers for other cancer types, we measured CD36 and CD47 expression in tumor microarrays (TMA) from multiple tumor types. Finally, we utilized a public database (Genomic Data Commons (GDC) Data Portal) from the National Cancer Institute (NCI) to map CD36 and CD47 expression in all reported clinical cases. Our findings support our hypothesis that CD36 and CD47 can be used as prognostic biomarkers for multiple cancer indications. Moreover, because of its biological MOA, patients with high expression of both CD36 and CD47 (dual high) may experience the greatest therapeutic benefit from VT1021 treatment.

## Patient response correlated to the CD36 and CD47 levels in tumors

NCT03364400 is a phase 1, first-in-human, multicenter, open-label, dose-escalation, and expansion study of VT1021 designed and sponsored by Vigeo Therapeutics, Inc. The escalation phase used a modified 3 + 3 design, and the primary outcomes are safety, tolerability, and pharmacokinetics in patients with advanced solid tumors. Patients were dosed twice weekly intravenously in 9 cohorts (0.5–15.6 mg/kg)^49^. In the expansion phase, subjects were enrolled into one of the five cohorts: recurrent glioblastoma (rGBM), pancreatic cancer, ovarian cancer, TNBC, and a high-CD36 and high-CD47 basket cohort. All patients received VT1021 (11.8 mg/kg, twice a week intravenously) until disease progression^50^. Since VT1021 showed clinical efficacy on some patient populations, it is urgent to specify the population that can benefit most from the VT1021 treatment.

**Fig. 1:**
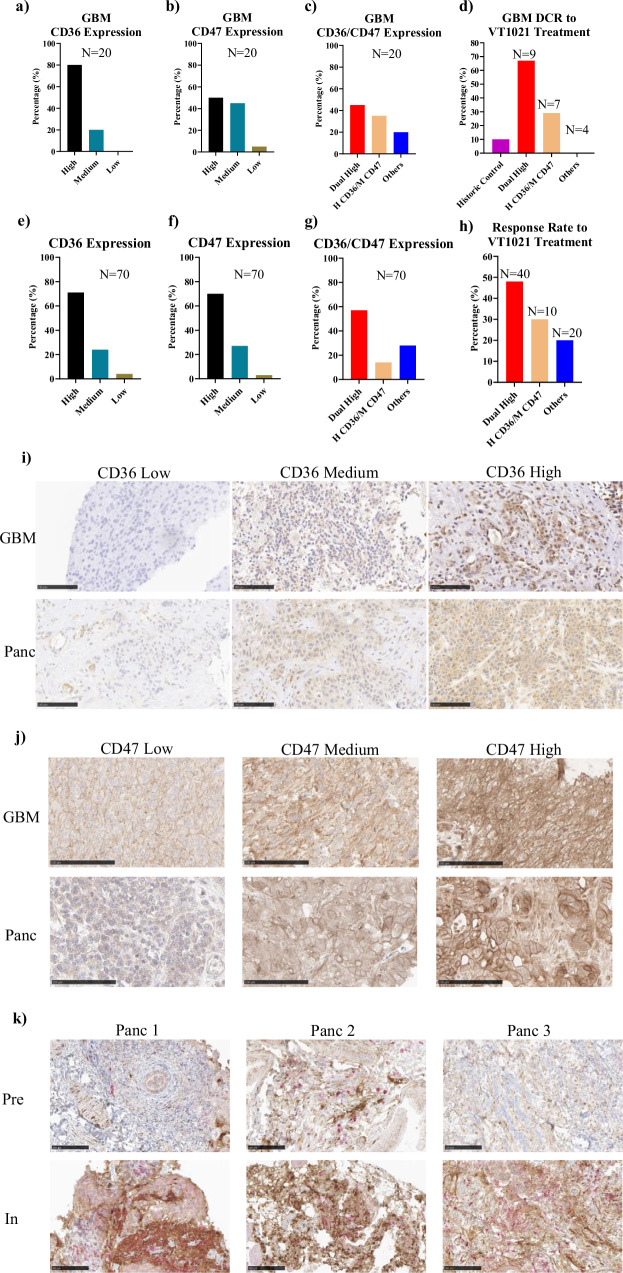
CD36 and CD47 signature in patients from VT1021 phase I trial. **a** Distribution of CD36 expression levels in rGBM cohort patients; **b** Distribution of CD47 expression levels in rGBM cohort patients; **c** Distribution of CD36 and CD47 expression levels in rGBM cohort patients; **d** DCR of rGBM cohort patients based on the CD36 and CD47 expression levels; **e** Distribution of CD36 expression levels in phase I patients; **f** Distribution of CD47 expression levels in phase I patients; **g** Distribution of CD36 and CD47 expression levels in phase I patients; **h** DCR of phase I patients based on the CD36 and CD47 expression levels; **i** Representative staining of different levels of CD36 expression in patient’s biopsies; **j** Representative staining of different levels of CD47 expression in patient’s biopsies. **k** Example of TSP-1 induction by VT1021 in paired biopsies from 3 pancreatic cancer patients. Black bar: high expression. Cyan bar: medium expression. Brown bar: low expression. Red bar: dual high expression of CD36 and CD47. Yellow bar: high expression of CD36 and medium expression of CD47. Blue bar: no dual high or high CD36 medium CD47. Purple bar: historic control^51^.

In the expansion phase, the rGBM cohort was the one that had the best disease control rate (DCR) among all 5 cohorts. In the rGBM cohort, the DCR was 45% (10 out of 22 evaluable patients), including 3 CR, 1 PR, and 6 SD with an average study duration of over 120 days. Since VT1021 stimulates TSP-1 expression in MDSCs, which then binds to CD36 and CD47 receptors in tumor cells to shrink the tumor^7^, the CD36 and CD47 levels of patient tumors were examined through IHC. We also developed a scoring system to distinguish different levels of CD36 and CD47 expression in tumor cells, based on the data from the escalation phase. Stained slides were scanned by Leica CS2 slide scanner and images with tumor cells were exported at 40X magnification using NDP.view2 (Hamamatsu Photonics). ImageJ-Fiji was used to measure the positive staining percentage and intensity for CD36 and CD47 expression, which is presented as the Optical Density (OD) value. In the escalation phase, biopsy samples were provided by 25 evaluable patients. All biopsies were stained and assigned an individual OD value. Subsequently, OD values were correlated with patient response to the VT1021 treatment. Based on this correlation, the cutoff value for high CD36 expression was set as greater than or equal to 0.125, and the cutoff value for low as less than 0.050. For CD47, the cutoff value for high expression was set as greater than or equal to 0.240, while the cutoff value for low as less than 0.150. As shown in Fig. 1a and Table 1, out of 20 evaluable patients who provided biopsies, 16 patients (80%) had high CD36 levels in the tumor cells, and 4 patients (20%) had medium CD36 levels in the tumor cells. None of the rGBM patients had low CD36 levels. This result is consistent with previous reports that rGBM patients have high CD36 expression^6,22^. For CD47, 10 patients (50%) had high CD47 levels in their tumor, 9 patients (45%) had medium CD47 levels, and 1 patient (5%) had low CD47 levels (Fig. 1b). These results indicate that the majority of rGBM patients have high CD47 expression in their tumors. Combining the CD36 and CD47 data, 9 patients (45%) had dual high CD36/CD47 levels, and 7 patients (35%) had high CD36 and medium CD47 (Fig. 1c). Furthermore, out of the 9 dual high patients, there were 3 patients with CR and 3 patients with SD, making the DCR 67% for these patients (Fig. 1d and Table 2). Out of the 7 patients with high CD36 and medium CD47, there were 2 patients with SD, for a DCR of 29%. For patients with neither dual high nor high CD36/medium CD47, 4 out of 4 had progressive disease. Compared to the historical response rate for rGBM patients (10%)^51^, our results suggest that rGBM patients with dual high levels of CD36 and CD47 or high CD36/medium CD47 will benefit the most from VT1021 treatment.

**Table 1:** CD36 and CD47 signatures of patients in the expansion rGBM cohort

	CD36 High	CD36 Medium	CD36 Low
CD47 High	9	1	0
CD47 Medium	7	2	0
CD47 Low	0	1	0

**Table 2:** DCR distribution with CD36/CD47 signature in the expansion rGBM cohort

	Total	Dual High	CD36 High / CD47 Medium	Others
CR	3	3	0	0
SD	5	3	2	0
PD	12	3	5	4
Total	20	9	7	4
DCR	40%	67%	29%	0%

Based on the findings described above, CD36 and CD47 could serve as effective dual markers to stratify patients and predict response for rGBM patients. Therefore, we have expanded this dual marker study to other indications in our phase I clinical trial. Including the rGBM cohort, 70 evaluable patients provided biopsies (escalation and expansion) (Table 3). Out of these 70 patients, 50 patients (71%) had high levels of CD36, 15 patients (24%) had medium levels and 3 patients (4%) had low levels in their tumor cells (Fig. 1e). For CD47, 49 patients (70%) had high levels, 19 patients (27%) had medium levels and 2 patients (3%) had low levels in their tumor cells (Fig. 1f). Combining the CD36 and CD47 data, 40 patients (57%) had dual high CD36/CD47 levels in their tumors, and 10 patients (14%) have high CD36 and medium CD47 (Fig. 1g). As Fig. 1h and Table 4 showed, out of the 40 dual high patients, there were 3 patients with CR and 16 with SD, for a DCR of 48%. Taking into account patients with high CD36 and medium CD47 (10 patients, 3 SD) the DCR for patients with high CD36 and high/medium CD47 is 44% (50 patients, 3 CR and 19 SD). For the remaining 20 patients that were neither dual high nor high CD36/medium CD47, there were 4 patients with SD, for a DCR of 20% (less than half the disease control rate of dual high or high CD36/medium CD47 patients). Figures 1i, j show representative staining of different levels of CD36 and CD47 in patients enrolled in the VT1021 phase I trial.

**Table 3:** CD36 and CD47 signatures of patients in both escalation and expansion phases

	CD36 High	CD36 Medium	CD36 Low
CD47 High	40	7	2
CD47 Medium	10	8	1
CD47 Low	0	2	0

**Table 4:** DCR distribution with CD36/CD47 signature in both escalation and expansion phases

	Total	Dual High	CD36 High / CD47 Medium	Others
CR	3	3	0	0
SD	23	16	3	4
PD	44	21	7	16
Total	70	40	10	20
DCR	37%	48%	30%	20%

Although in-treatment biopsies are not part of standard procedures in GBM clinical trials, due to the nature of GBM disease, it has been well established by our previous published reports that TSP-1 expression is increased in myeloid cells in the circulation of GBM patients treated with VT1021^50^. We also have demonstrated that increased expression of TSP-1 in circulating myeloid cells correlates with increased expression of TSP-1 in tumor tissues of other tumor types^49,50^. Here we show examples of TSP-1 induction by VT1021 in the paired biopsies of 3 pancreatic cancer patients from the expansion cohort (Fig. 1k). After VT1021 treatment, both TSP-1 (brown staining) and TSP-1 positive MDSCs (brown and pink staining) were significantly increased in the in-treatment biopsies.

Despite patients being at the late stages of their disease and exposed to multiple lines of treatment, the DCR to the VT1021 treatment still achieved an impressive 50% in dual high populations. It is thus reasonable to infer that the DCR will increase if patients can be treated with VT1021 at earlier stages of the disease. These findings validate that CD36 and CD47 dual markers could be used to select patient populations for the VT1021 treatment.

## CD36 and CD47 levels are increased in multiple cancers through TMAs

**Fig. 2:**
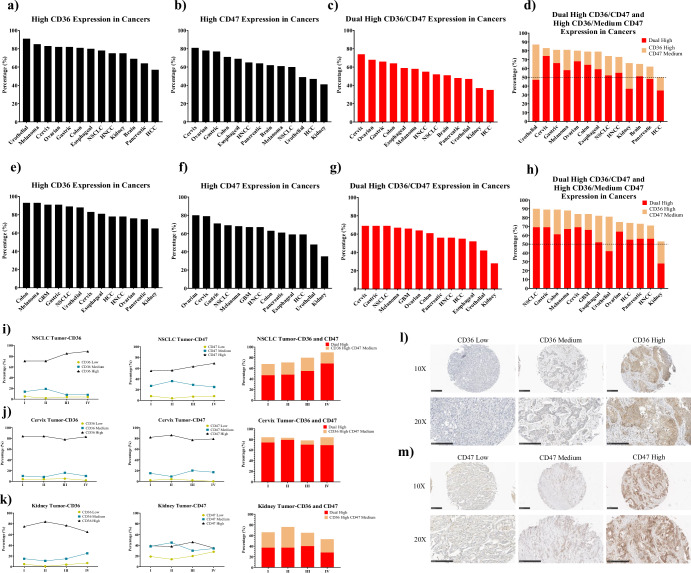
CD36 and CD47 signature in TMAs from 13 types of indications. **a** Percentage of cases with high CD36 expression in TMAs of all stages; **b** Percentage of cases with high CD47 expression in TMAs of all stages; **c** Percentage of cases with dual high expression of CD36 and CD47 in TMAs of all stages; **d** Percentage of cases with dual high expression of CD36/CD47 and cases with high CD36/medium CD47 expression in TMAs of all stages, black dot line indicates 50%; **e** Percentage of cases with high CD36 expression in TMAs of Stage IV; **f** Percentage of cases with high CD47 expression in TMAs of Stage IV; **g** Percentage of cases with dual high expression of CD36 and CD47 in TMAs of Stage IV; **h** Percentage of cases with dual high expression of CD36/CD47 and cases with high CD36/medium CD47 expression in TMAs of Stage IV, black dot line indicates 50%; **i**–**k** CD36 and CD47 expression pattern in TMAs of NSCLC (**i**), Cervix Cancer (**j**), and Kidney Cancer (**k**), left panel: change of CD36 expression in different stages, middle panel: change of CD47 expression in different stages, right panel: percentage of cases with dual high expression of CD36/CD47 and cases with high CD36/medium CD47 expression in different stages; **l** representative staining of different levels of CD36 expression in TMAs; **m** representative staining of different levels of CD47 expression in TMAs. Black bar: high expression. Red bar: dual high expression of CD36 and CD47. Yellow bar: high expression of CD36 and medium expression of CD47. Black triangle: high expression. Cyan square: medium expression. Brown dot: low expression.

The above studies were based on the VT1021 phase I clinical trial and focused mainly on rGBM, Pancreatic Cancer, and Ovarian Cancer. To further expand the use of CD36/CD47 dual markers as a patient population selective tool, TMAs from multiple cancers were purchased and stained histologically to examine CD36 and CD47 expression. Our analysis of TMAs from 13 different cancer indications revealed that all were comprised of over 60% of patients with high CD36, with the exception of Hepatocellular carcinoma (HCC), which was comprised of 57% of patients with high CD36 expression (Fig. 2a and Table 5). Similarly, 10/13 indications were comprised of over 60% of patients with high levels of CD47 with the remaining 3 indications comprised of over 40% CD47 high cases (Fig. 2b). When considering patients that were high for both CD36 and CD47, 11 out of 13 indications had more than 47% of cases with dual high levels of CD36/CD47, with Kidney Cancer (37%) and HCC (35%) as the exception (Fig. 2c). Since patients with high CD36 and medium CD47 also showed clinical benefits from the VT1021 treatment, it was relevant to include this population in the analysis: 12 out of 13 indications had more than 60% of cases with either dual high levels of CD36/CD47 or high CD36/medium CD47, with HCC (50%) as the only exception (Fig. 2d).

**Table 5:** CD36 and CD47 signatures of TMAs at all stages

Indications	Cases	CD36 Low	CD36 Medium	CD36 High	CD47 Low	CD47 Medium	CD47 High	CD36 High CD47 Medium	Dual High
Brain	260	3%	28%	69%	6%	31%	62%	14%	51%
Cervix	408	4%	11%	83%	2%	15%	81%	9%	74%
Colon	242	3%	10%	81%	5%	19%	71%	15%	64%
Esophageal	259	3%	14%	80%	5%	25%	69%	20%	59%
Gastric	199	2%	10%	82%	3%	18%	77%	15%	66%
HCC	418	15%	27%	57%	16%	33%	47%	15%	35%
HNCC	241	5%	17%	75%	8%	27%	65%	18%	55%
Kidney	369	5%	17%	75%	20%	36%	41%	29%	37%
Melanoma	202	1%	8%	85%	5%	27%	61%	23%	58%
NSCLC	305	4%	12%	78%	7%	29%	60%	22%	52%
Ovarian	366	2%	11%	82%	2%	16%	78%	12%	68%
Pancreatic	182	7%	33%	64%	4%	29%	64%	14%	48%
Urothelial	80	2%	8%	91%	9%	42%	49%	40%	47%

Although routine cancer screenings, such as mammograms and colonoscopy, allow breast cancer and colon cancer patients to be diagnosed at the early stage of the disease, most cancer patients, when first diagnosed, are already at an advanced stage of their diseases. To investigate the CD36 and CD47 signatures of patients with late-stage diseases, further studies were performed on the TMA data, focusing on stage IV cases. Among the 13 different indications we examined, over 75% of patients with stage IV disease had high CD36 levels in all indications except Kidney cancer (65%) (Fig. 2e and Table 6). Similarly, over 48% of patients in all indications except kidney cancer (35%) had high CD47 expression (Fig. 2f). Combining CD36 and CD47, in 11 out of 13 indications greater than 50% patients with stage IV disease had dual high levels of CD36/CD47 (Fig. 2g), and in 12 out of 13 indications greater than 70% of stage IV patients had either dual high levels of CD36/CD47 or high CD36/medium CD47 (Fig. 2h).

**Table 6:** CD36 and CD47 signatures of TMAs at stage IV

Indications	Cases	CD36 Low	CD36 Medium	CD36 High	CD47 Low	CD47 Medium	CD47 High	CD36 High CD47 Medium	Dual High
Brain	63	0%	9%	91%	10%	24%	67%	18%	66%
Cervix	16	2%	10%	83%	0%	17%	79%	15%	69%
Colon	66	1%	5%	93%	6%	32%	63%	28%	61%
Esophageal	9	0%	19%	81%	4%	37%	59%	30%	52%
Gastric	25	1%	7%	91%	5%	23%	71%	20%	69%
HCC	96	6%	16%	78%	11%	29%	59%	19%	55%
HNCC	64	2%	12%	78%	4%	22%	67%	15%	56%
Kidney	41	7%	25%	65%	28%	34%	35%	25%	28%
Melanoma	59	0%	7%	93%	6%	27%	68%	21%	67%
NSCLC	18	4%	8%	89%	8%	25%	69%	21%	69%
Ovarian	33	3%	15%	76%	1%	16%	80%	11%	64%
Pancreatic	24	0%	17%	75%	0%	29%	61%	17%	56%
Urothelial	11	3%	9%	88%	12%	39%	48%	39%	42%

To further understand if CD36/CD47 levels were correlated with disease progression, we further analyzed the TMA data by comparing the expression levels of CD36/CD47 across different stages for each indication (Fig. 2i–k). As indicated in Table 7, these indications could be divided into 3 categories: high CD36/high CD47 (dual-high), high CD36/medium CD47, and others. In some indications, such as Brain cancer and Non-Small Cell Lung Cancer (NSCLC), the CD36/CD47 expression levels were positively correlated with disease progression (Fig. 2i). There were also indications in which the CD36/CD47 expression levels were consistent across all stages, such as Cervical cancer and Ovarian cancer (Fig. 2j). Finally, some indications, such as Kidney cancer and Melanoma, had no distinct pattern of CD36/CD47 expression levels across different disease stages (Fig. 2k). These data suggest that different indications might have different levels of dependency on CD36/CD47 for disease progression and tumor survival. Patients with indications with positive correlation or stable levels of CD36/CD47 across disease stages might benefit more from VT1021 treatment. Figure 2l, m show representative staining of different levels of CD36 and CD47 in TMAs.

**Table 7:** CD36 and CD47 signatures of TMAs at different stages

Indications	Stage	Cases	CD36 High	CD47 High	CD36 High CD47 Medium	Dual High
Brain	1	62	62%	55%	11%	45%
Brain	2	54	65%	69%	11%	53%
Brain	3	52	72%	66%	16%	55%
Brain	4	63	91%	67%	18%	66%
Cervix	I	294	84%	82%	10%	74%
Cervix	II	49	84%	86%	4%	79%
Cervix	III	47	78%	77%	8%	70%
Cervix	IV	16	83%	79%	15%	69%
Colon	I	24	86%	90%	6%	81%
Colon	II	113	75%	74%	9%	63%
Colon	III	39	76%	68%	10%	59%
Colon	IV	66	93%	63%	28%	61%
Esophageal	I	26	68%	76%	12%	56%
Esophageal	II	86	74%	69%	21%	54%
Esophageal	III	138	86%	68%	20%	64%
Esophageal	IV	9	81%	59%	30%	52%
Gastric	I	34	77%	77%	15%	62%
Gastric	II	76	76%	84%	9%	66%
Gastric	III	45	84%	69%	18%	66%
Gastric	IV	25	91%	71%	20%	69%
HCC	I	30	33%	36%	4%	22%
HCC	II	158	51%	51%	13%	33%
HCC	III	134	55%	36%	16%	25%
HCC	IV	96	78%	59%	19%	55%
HNCC	I	16	67%	77%	10%	54%
HNCC	II	92	67%	58%	17%	47%
HNCC	III	69	85%	70%	19%	64%
HNCC	IV	64	78%	67%	15%	56%
Kidney	I	160	75%	39%	29%	37%
Kidney	II	75	84%	38%	39%	37%
Kidney	III	59	77%	46%	25%	40%
Kidney	IV	41	65%	35%	25%	28%
Melanoma	I	11	73%	64%	23%	52%
Melanoma	II	87	84%	57%	26%	54%
Melanoma	III	15	100%	82%	16%	82%
Melanoma	IV	59	93%	68%	21%	67%
NSCLC	I	69	71%	55%	21%	47%
NSCLC	II	82	71%	56%	23%	48%
NSCLC	III	136	85%	63%	25%	55%
NSCLC	IV	18	89%	69%	21%	69%
Ovarian	I	257	83%	77%	13%	69%
Ovarian	II	49	84%	79%	15%	65%
Ovarian	III	26	74%	87%	6%	65%
Ovarian	IV	33	76%	80%	11%	64%
Pancreatic	I	55	67%	61%	17%	50%
Pancreatic	II	91	59%	67%	11%	45%
Pancreatic	III	12	58%	58%	11%	47%
Pancreatic	IV	24	75%	61%	17%	56%
Urothelial	I	9	96%	44%	48%	44%
Urothelial	II	32	94%	44%	47%	44%
Urothelial	III	28	87%	56%	30%	52%
Urothelial	IV	11	88%	48%	39%	42%

Overall, these TMA data indicate that multiple types of tumors expressed high levels of CD36/CD47, and that these patients may benefit from VT1021 treatment. Importantly, these results highlight the potential of implementing CD36/CD47 as dual markers to select patients for VT1021 treatment in a broad range of cancer indications.

## CD36 and CD47 gained copy numbers in multiple cancers through the NCI database

**Fig. 3:**
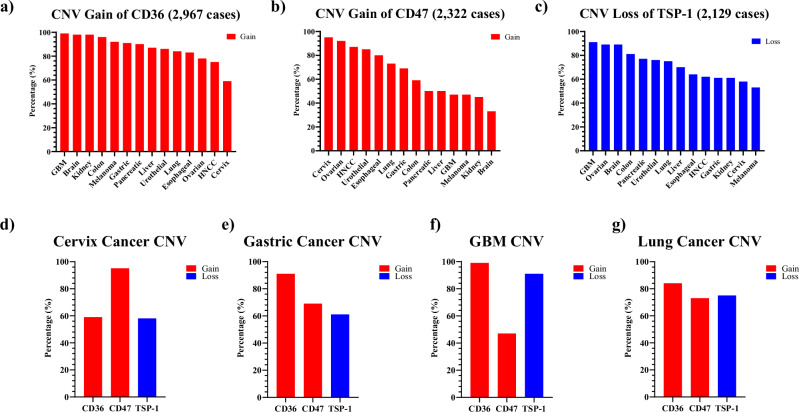
Patient’s copy number variation (CNV) of CD36, CD47, and TSP-1 from NCI GDC database. **a** Percentage of cases that gained CD36 copy number in 13 types of cancer; **b** Percentage of cases that gained CD47 copy number in 13 types of cancer; **c** Percentage of cases that lost TSP-1 copy number in 13 types of cancer; **d**–**g** Percentage of cases that had CD36, CD47, and TSP-1 copy number changes in Cervix Cancer (**d**), Gastric Cancer (**e**), GBM (**f**), and Lung Cancer (**g**). Red bar: gain copy number. Blue bar: loss copy number.

To augment the significance of the TMA data, we turned to a public database of reported patient genomic data. NCI GDC Data Portal is a repository and computational platform, containing high-quality datasets of cancer genomic studies spanning over 40,000 cases^52,53^. In this database, across the 13 indications we studied, 2967 cases had reported CD36 copy number variation (CNV) data and 2322 cases with reported CD47 CNV data (Table 8). Our analysis revealed that >59% of cases possessed gain of copy number of CD36, with Brain cancer and Kidney cancer the highest at 98% (Fig. 3a). Moreover, 12 out of 13 indications had >45% of cases with gain of copy number for CD47, with Cervix cancer having the highest percentage (95%) (Fig. 3b). These data support our histological findings that CD36 and CD47 are over-expressed in multiple types of cancers.

**Table 8:** CD36, CD47 and TSP-1 signatures in NCI GDC database

Indication	CD36 Total Cases	Cases Gain CD36 copy number	Percentage of Cases Gain CD36 copy number	CD47 Total Cases	Cases Gain CD47 copy number	Percentage of Cases Gain CD47 copy number	TSP-1 Total Cases	Cases Loss TSP-1 copy number	Percentage of Cases Loss TSP-1 copy number
Brain	532	524	98%	102	34	33%	197	176	89%
GBM	412	406	99%	62	29	47%	135	123	91%
Cervix	71	42	59%	150	142	95%	99	57	58%
Colon	219	210	96%	91	54	59%	174	141	81%
Esophageal	101	84	83%	96	77	80%	69	44	64%
Gastric	200	181	91%	125	86	69%	145	89	61%
Liver	115	100	87%	62	31	50%	77	54	70%
HNCC	185	138	75%	255	223	87%	164	101	62%
Kidney	323	318	98%	263	118	45%	106	65	61%
Melanoma	242	223	92%	141	66	47%	192	102	53%
Lung	495	415	84%	538	393	73%	440	332	75%
Ovarian	276	216	78%	299	276	92%	288	256	89%
Pancreatic	40	36	90%	30	15	50%	43	33	77%
Urothelial	168	144	86%	170	144	85%	135	102	76%

In addition, since VT1021 stimulates TSP-1 expression, which binds to CD36/CD47 on the surface of tumor cells, we also investigated the TSP-1 CNV in the NCI database. Not surprisingly, across all indications more than 50% of cases per indication had a loss of copy number of TSP-1 (Fig. 3c). We next sought to determine whether there was a correlation between the gain of copy number for CD36/CD47 and loss of copy number for TSP-1. Specifically, we focused on the indications with the highest percentage of high CD36/CD47 patients. As expected, these indications all had a high percentage of copy number gain for CD36 and CD47 and loss of copy number for TSP-1 (Fig. 3d–g). Cervix cancer cases reported 59% cases gained CD36 copy number, 95% cases gained CD47 copy number, and 58% of cases lost TSP-1 copy number. In gastric cancer cases reported 91% cases gained CD36 copy number, 69% cases gained CD47 copy number, and 61% cases lost TSP-1 copy number. GBM cases reported 99% cases gained CD36 copy number, 47% cases gained CD47 copy number, and 91% cases lost TSP-1 copy number. In lung cancer cases reported 84% cases gained CD36 copy number, 73% cases gained CD47 copy number, and 75% cases lost TSP-1 copy number.

This analysis of the NCI GDC database supports our previous findings and indicates that the CD36/CD47 dual marker can be broadly used to select patients for VT1021 by cancer indication and progression.

VT1021 is a first-in-class therapeutic agent that has completed both dose escalation and indication expansion clinical trials^49,50^. VT1021 is currently being tested in a phase II/III clinical study in GBM. In this report, we identified CD36 and CD47 as potential biomarkers to select patients for VT1021 treatment. Specifically, we found that CD36 and CD47 were ubiquitously expressed in tumor cells across virtually all types of cancers evaluated in this study. Critically, both biomarkers are essential for the survival of tumor cells^54,55^. High levels of either CD36 or CD47 are associated with poor outcomes for cancer patients^46,47^. Due to their ubiquitous expression and association with poor outcomes, the dual marker of CD36 and CD47 is an ideal selection criterion for VT1021 treatment or other TSP-1-based therapeutic strategies.

In the phase I trial of VT1021, the overall DCR of patients with dual high CD36 and CD47 was 48%, including 3 CR. For patients with high CD36 and medium CD47, across all indications, the DCR was ~30%. These results indicate that the CD36/CD47 dual markers provide potential selection criteria for VT1021 treatment. Notably, a small percentage of patients with dual high CD36/CD47 did not respond to the VT1021 treatment. While no single parameter emerged to account for lack of response by these patients, there are multiple variables that currently confound this analysis, such as prior lines of treatment, and tumor heterogeneity^56^. While most patients had uniform expression of CD36 and CD47, we did find that some tumors were comprised of cells that had different levels of CD36 and CD47 expression. We hypothesize that when patients with heterogeneous tumor cell expression of CD36/47 are treated with VT1021, tumor cells with high CD36 and CD47 would be killed, but tumor cells with low levels of CD36 and CD47 would not respond to the VT1021 treatment, resulting in a lack of response. For these situations, combination therapy may be a more efficacious strategy and we are currently investigating in that hypothesis.

Our TMA data supports that tumor cells of patients with late stage disease express high levels of CD36 and CD47. These patients are generally the most difficult to treat and have the lowest response rates to existing therapies. Accordingly, VT1021 may be an excellent option for patients with late-stage disease as either a single agent or in combination with other therapeutic modalities. Taken together, we identified CD36 and CD47 as dual biomarkers to be used as prognostic biomarkers for multiple cancers. Our findings predict that patients with dual high CD36 and CD47 would benefit the most from the VT1021 treatment.

## Methods

### Clinical trial and patient sample analysis

Patient biopsy samples used in this study were obtained from the clinical trial (NCT03364400), designed and sponsored by Vigeo Therapeutics, Inc. This trial was a phase 1 study of VT1021 treatment in multiple advanced solid tumor types, including both escalation and expansion phases. There were 28 patients enrolled in the escalation phase (25 patients provided biopsies) and 47 patients enrolled in the expansion phase (45 patients provided biopsies). Clinical response was evaluated by CT or MRI every 8 weeks ± 1 week according to RECIST guidelines (version 1.1) and iRECIST guidelines, or RANO guidelines for rGBM patients with iRANO modifications. Clinical benefit was defined by best overall response with complete response (CR), partial response (PR), or stable disease (SD) > 2 cycles (8 weeks). For clinical response and biomarker studies reported here, a subject ID number is assigned to each patient.

### Immunohistochemistry (IHC) staining

For patient biopsies, either a pre-treatment biopsy or an archival tumor specimen is considered the Screen biopsy. The in-treatment (In-Tx) biopsies were collected at the end of Cycle 1 Week 4, or at any time during Cycle 2, or thereafter at the discretion of the Investigator.

Biopsies were stained with either CD36 or CD47 for single staining. The paraffin-sectioned slides were deparaffinized with xylene and rehydrated in decreasing concentration of ethanol to water. The antigen retrieval was performed with Tris-EDTA buffer (pH 9.0, Abcam ab93684) at 110 °C for 17 mins. The slides were then blocked with 2.5% goat serum (Vector Laboratories) for 30 min at room temperature. Slides were incubated overnight at 4 °C with either rabbit anti-CD36 primary antibody (EPR6573, ab133625, Abcam) or rabbit anti-CD47 primary antibody (SP279, ab226837, Abcam). The slides were then washed in PBS with 0.05% Tween (three times for 5 min), followed by incubation with horseradish peroxidase (HRP) conjugated secondary antibody (Vector Laboratories) for 30 min at room temperature. Slides were then incubated with DAB substrate (Vector Laboratories) followed by counterstain with Hematoxylin (Vector Lab, H3401).

Biopsies were stained with antibodies against TSP-1 and CD11b for double staining. Following the single staining protocol mentioned above, slides were stained with rabbit anti-TSP-1 primary antibody (EPR22927-54, ab267397, Abcam) first. After the slides finished incubation with DAB substrate, the slides were washed in PBS with 0.05% Tween to eliminate the substrate reaction. Then the antigen retrieval was performed again with Citrate buffer (pH 6.0, Abcam ab64214) at 110 °C for 2 mins. The slides were then blocked with 2.5% horse serum (Vector Laboratories) for 30 min at room temperature. Slides were incubated overnight at 4 °C with horse anti-CD11b primary antibody (EPR1344, ab133357, Abcam). The slides were then washed in TBS with 0.05% Tween (three times for 5 min), followed by incubation with alkaline phosphatase (AP) conjugated secondary antibody (Vector Laboratories) for 30 min at room temperature. Slides were then incubated with Warp Red substrate (Biocare Medical) followed by counterstain with Hematoxylin (Vector Lab, H3401).

### TMAs analysis

All TMA slides were purchased from TissueArray.Com LLC (US Biomax, Inc), and stained with either CD36 or CD47, following the protocol mentioned above.

### NCI database

The public NCI database was accessed through the NCI GDC data portal (https://portal.gdc.cancer.gov/). Both case number and copy number variation (CNV) data were extracted from the database and then graphed by GraphPad Prism.

### Statistical analysis

The disease control rate (DCR) used for clinical outcomes was calculated as the percentage of subjects with advanced cancer whose therapeutic intervention has led to a complete response, partial response, or stable disease. All bar graphs and scatter plots with bar (individual dot representing a patient) showed the mean and standard error of the mean (SEM) and were generated with Graphpad Prism 9.3.1.

## Data Availability

The datasets generated during and/or analyzed during the current study are not publicly available due to the clinical and confidential nature of the material but can be made available from the corresponding author on reasonable request.
